# Supplementary material for: Estimated quantity of swine virus genomes based on quantitative PCR analysis in spray-dried porcine plasma samples collected from multiple manufacturing plants
Source: PLoS One. 2022 May 23;17(5):e0259613. doi: 10.1371/journal.pone.0259613 (PMC9126402; doi:10.1371/journal.pone.0259613)
Supplement: Attachment — Submitted filename: PONE-D-21-33668_reviewer.pdf [file pone.0259613.s003.pdf]

PLOS ONE

Estimated quantity of swine virus genomes based on quantitative PCR analysis in spray-dried porcine plasma samples collected from multiple manufacturing plants.

--Manuscript Draft--

**Manuscript Number:**	PONE-D-21-33668
**Article Type:**	Research Article
**Full Title:**	Estimated quantity of swine virus genomes based on quantitative PCR analysis in spray-dried porcine plasma samples collected from multiple manufacturing plants.
**Short Title:**	Quantitation of viral genomes in porcine plasma collected from abattoirs
**Corresponding Author:**	Francisco Javier Polo Pozo, Ph.D.<br>APC EUROPE, S.L.U.<br>barbera del valles, Barcelona SPAIN
**Keywords:**	spray-dried porcine plasma<br>swine viruses<br>qPCR<br>Worldwide viruses distribution
**Abstract:**	This survey was conducted to estimate the incidence and level of potential viral contamination in commercially collected porcine plasma. Samples of spray dried porcine plasma (SDPP) were collected over a 12- month period from eight spray drying facilities in Spain, England, Northern Ireland, Brazil, Canada, and the United States. In this survey, viral load for several porcine pathogens including SVA, TGEV, PRRSV (EU and US strains), PEDV, PCV2, SIV, SDCoV and PPV were determined by qPCR. Regression of Ct on TCID50 of serial diluted stock solution of each virus allowed the estimate of potential viral level in SDPP and unprocessed liquid plasma (using typical solids content of commercially collected porcine plasma). In this survey SVA, TGEV or SDCoV were not detected in any of the SDPP samples. Brazil SDPP samples were free of PRRSV and PEDV. Samples of SDPP from North America primarily contained the PRRSV-US strain while the European samples contained the PRRSV-EU strain (except for one sample from each region containing a low estimated level of the alternative PRRSV strain). Estimated viral level tended to be low ranging from <1.0 $\log_{10}$ $TCID_{50}$ to <2.5 $\log_{10}$ $TCID_{50}$. Estimated level of SIV was the exception with a very low incidence rate but higher estimated viral load <3.9 $\log_{10}$ $TCID_{50}$. In summary, the incidence of potential viral contamination in commercially collected porcine plasma was variable and estimated virus level in samples containing viral DNA/RNA was low.
**Order of Authors:**	Elena Blázquez, PhD
	Joan Pujols, PhD
	Segalés Joaquim, PhD
	Carmen Rodríguez
	Joy M. Campbell, PhD
	Louis E. Russell, PhD
	Francisco Javier Polo Pozo, Ph.D.
**Additional Information:**	
**Question**	**Response**
**Financial Disclosure**<br><br>Enter a financial disclosure statement that describes the sources of funding for the work included in this submission. Review the submission guidelines for detailed	Funding for this study was provided by APC Europe, S.L.U., Granollers, Spain, and APC LLC, Ankeny, IA, 50021, USA. These companies manufacture animal blood products for animal consumption. The funders provided support in the form of salaries for authors EB, CR, JC, LR and JPolo, but did not have any additional role in the study design, data collection and analysis, decision to publish, or preparation of the manuscript. The specific roles of these authors are articulated in the ‘author

Powered by Editorial Manager® and ProduXion Manager® from Aries Systems Corporation

requirements. View published research articles from *PLOS ONE* for specific examples.<br><br>This statement is required for submission and **will appear in the published article** if the submission is accepted. Please make sure it is accurate.<br><br>**Unfunded studies**<br>Enter: *The author(s) received no specific funding for this work.*<br><br>**Funded studies**<br>Enter a statement with the following details:<br>• Initials of the authors who received each award<br>• Grant numbers awarded to each author<br>• The full name of each funder<br>• URL of each funder website<br>• Did the sponsors or funders play any role in the study design, data collection and analysis, decision to publish, or preparation of the manuscript?<br>• **NO** - Include this sentence at the end of your statement: *The funders had no role in study design, data collection and analysis, decision to publish, or preparation of the manuscript.*<br>• **YES** - Specify the role(s) played.<br><br>* typeset	contributions' section.
**Competing Interests**<br><br>Use the instructions below to enter a competing interest statement for this submission. On behalf of all authors, disclose any competing interests that could be perceived to bias this work—acknowledging all financial support and any other relevant financial or non-financial competing interests.<br><br>This statement is required for submission and **will appear in the published article** if the submission is accepted. Please make sure it is accurate and that any funding sources listed in your Funding Information later in the submission form are also declared in your Financial Disclosure	The authors have read the journal's policy and the authors of this manuscript have the following competing interests: EB, CR, and JPolo are employed by APC Europe, S.L.U. Granollers, Spain and JC, LR and JPolo are employed by APC LLC, Ankeny, IA, USA. APC Europe and APC LLC manufactures and sells spray-dried animal plasma; however, the companies did not have any additional role in the study design, data collection and analysis, decision to publish, or preparation of the manuscript. This does not alter the authors' adherence to all PLOS ONE policies on sharing data and materials. JPujols, and JS declared no conflict of interest.

Powered by Editorial Manager® and ProduXion Manager® from Aries Systems Corporation

statement.<br><br>View published research articles from *PLOS ONE* for specific examples.<br><br>**NO authors have competing interests**<br><br>Enter: *The authors have declared that no competing interests exist.*<br><br>**Authors with competing interests**<br><br>Enter competing interest details beginning with this statement:<br><br>*I have read the journal's policy and the authors of this manuscript have the following competing interests: [insert competing interests here]*<br><br>* typeset	
**Ethics Statement**<br><br>Enter an ethics statement for this submission. This statement is required if the study involved:<br><br>• Human participants<br>• Human specimens or tissue<br>• Vertebrate animals or cephalopods<br>• Vertebrate embryos or tissues<br>• Field research<br><br>Write "N/A" if the submission does not require an ethics statement.<br><br>General guidance is provided below. Consult the submission guidelines for detailed instructions. **Make sure that all information entered here is included in the Methods section of the manuscript.**	No animal or human research has been associated with this research

**Format for specific study types**<br><br>**Human Subject Research (involving human participants and/or tissue)**<br>• Give the name of the institutional review board or ethics committee that approved the study<br>• Include the approval number and/or a statement indicating approval of this research<br>• Indicate the form of consent obtained (written/oral) or the reason that consent was not obtained (e.g. the data were analyzed anonymously)<br><br>**Animal Research (involving vertebrate animals, embryos or tissues)**<br>• Provide the name of the Institutional Animal Care and Use Committee (IACUC) or other relevant ethics board that reviewed the study protocol, and indicate whether they approved this research or granted a formal waiver of ethical approval<br>• Include an approval number if one was obtained<br>• If the study involved *non-human primates*, add *additional details* about animal welfare and steps taken to ameliorate suffering<br>• If anesthesia, euthanasia, or any kind of animal sacrifice is part of the study, include briefly which substances and/or methods were applied<br><br>**Field Research**<br><br>Include the following details if this study involves the collection of plant, animal, or other materials from a natural setting:<br>• Field permit number<br>• Name of the institution or relevant body that granted permission	
**Data Availability**<br><br>Authors are required to make all data underlying the findings described fully available, without restriction, and from the time of publication. PLOS allows rare exceptions to address legal and ethical concerns. See the PLOS Data Policy and FAQ for detailed information.	Yes - all data are fully available without restriction

A Data Availability Statement describing where the data can be found is required at submission. Your answers to this question constitute the Data Availability Statement and **will be published in the article**, if accepted.<br><br>**Important:** Stating 'data available on request from the author' is not sufficient. If your data are only available upon request, select 'No' for the first question and explain your exceptional situation in the text box.<br><br>Do the authors confirm that all data underlying the findings described in their manuscript are fully available without restriction?	
**Describe where the data may be found in full sentences. If you are copying our sample text, replace any instances of XXX with the appropriate details.**<br><br>• If the data are **held or will be held in a public repository**, include URLs, accession numbers or DOIs. If this information will only be available after acceptance, indicate this by ticking the box below. For example: *All XXX files are available from the XXX database (accession number(s) XXX, XXX.*).<br>• If the data are all contained **within the manuscript and/or Supporting Information files**, enter the following: *All relevant data are within the manuscript and its Supporting Information files.*<br>• If neither of these applies but you are able to provide **details of access elsewhere**, with or without limitations, please do so. For example:<br><br>*Data cannot be shared publicly because of [XXX]. Data are available from the XXX Institutional Data Access / Ethics Committee (contact via XXX) for researchers who meet the criteria for access to confidential data.*<br><br>*The data underlying the results presented in the study are available from (include the name of the third party*	All relevant data are within the paper and its Supporting information files.

and contact information or URL).<br>• This text is appropriate if the data are owned by a third party and authors do not have permission to share the data.<br><br>* typeset	
Additional data availability information:	

Manuscript

Click here to access/download;Manuscript;Quantification of different swine virus presence in SDPP.docx

Estimated quantity of swine virus genomes based on quantitative PCR analysis in spray-dried porcine plasma samples collected from multiple manufacturing plants.

Elena Blázquez[1,2,3*], Joan Pujols[1,3], Joaquim Segalés[3,4,5], Carmen Rodríguez[2], Joy Campbell[6], Louis Russell[6], Javier Polo[2,6,*]

Short title: Quantitation of viral genomes in porcine plasma collected from abattoirs

[1] IRTA, Centre de Recerca en Sanitat Animal (CReSA-IRTA), Bellaterra, Barcelona, Spain

[2] APC EUROPE S.L.U., Granollers, Barcelona, Spain

[3] OIE Collaborating Centre for the Research and Control of Emerging and Reemerging Swine Diseases in Europe (IRTA-CReSA), Bellaterra, Barcelona, Spain

[4] Departament de Sanitat i Anatomia Animals, Universitat Autònoma de Barcelona (UAB), Bellaterra, Barcelona, Spain

[5] UAB, Centre de Recerca en Sanitat Animal (CReSA, IRTA-UAB), Campus de la Universitat Autònoma de Barcelona, Bellaterra, Barcelona, Spain

[6] APC LLC, Ankeny, Iowa, United States of America

* Corresponding author: javier.polo@apc-europe.com (JPolo)

Abstract (max 300 words)

This survey was conducted to estimate the incidence and level of potential viral contamination in commercially collected porcine plasma. Samples of spray dried porcine plasma (SDPP) were collected over a 12- month period from eight spray drying facilities in Spain, England, Northern Ireland, Brazil, Canada, and the United States. In this survey, viral load for several porcine pathogens including SVA, TGEV, PRRSV (EU and US strains), PEDV, PCV2, SIV, SDCoV and PPV were determined by qPCR. Regression of Ct on $TCID_{50}$ of serial diluted stock solution of each virus allowed the estimate of potential viral level in SDPP and unprocessed liquid plasma (using typical solids content of commercially collected porcine plasma). In this survey SVA, TGEV or SDCoV were not detected in any of the SDPP samples. Brazil SDPP samples were free of PRRSV and PEDV. Samples of SDPP from North America primarily contained the PRRSV-US strain while the European samples contained the PRRSV-EU strain (except for one sample from each region containing a low estimated level of the alternative PRRSV strain). Estimated viral level tended to be low ranging from <1.0 $\log_{10}$ $TCID_{50}$ to <2.5 $\log_{10}$ $TCID_{50}$. Estimated level of SIV was the exception with a very low incidence rate but higher estimated viral load <3.9 $\log_{10}$ $TCID_{50}$. In summary, the incidence of potential viral contamination in commercially collected porcine plasma was variable and estimated virus level in samples containing viral DNA/RNA was low.

Keywords; spray-dried porcine plasma; swine viruses; qPCR; Worldwide viruses distribution

Introduction

Spray dried porcine plasma (SDPP) is a complex mixture of functional components including immunoglobulins, albumin, transferrin, fibrinogen, lipids, growth factors, bioactive peptides, enzymes, hormones, and amino acids commonly used in feed for young animals including pigs, calves, and poultry [1-4].

It has been speculated that the use of SDPP in swine feed contributed to the spread of infective viruses such as *Porcine circovirus 2* (PCV-2) and *Porcine epidemic diarrhea virus* (PEDV) [5-7]. However, other evidence demonstrates that reduced mortality and morbidity is associated with the use of SDPP in pig diets [1, 3, 8, 9] and experimental and epidemiological evidence demonstrate that SDPP does not spread diseases [10-12].

The manufacturing process to produce SDPP includes multiple hurdles that have been validated to inactivate potential viral contamination. These hurdles include spray drying (SD, 80°C throughout substance), ultraviolet light (UV) treatment (3000 J/L) and post drying storage (PDS) at 20°C for 14 d [13-19]. Depending on the virus, the theoretical cumulative inactivation for SD and PDS range from 5.8 to 9.1 log_{10} $TCID_{50}$/g liquid plasma, while SD, PDS and UV range from 11.7 to 20.9 log_{10} $TCID_{50}$/g liquid plasma (Table 1). The World Health Organization recommended cumulative robust inactivation procedures capable to eliminate 4 log_{10} of virus by each of these steps in the manufacturing process for human blood and plasma products [20, 21].

While the inactivation capacity of the multiple hurdle manufacturing process has been validated for several economically important swine viruses, it is also important to estimate the potential virus quantity in liquid plasma used to produce SDPP. Therefore, this

survey was conducted to estimate the quantity and determine the frequency of genome detection of different swine viruses in commercially produced SDPP samples collected from 8 different manufacturing plants. Results obtained from quantitative polymerase chain reaction (qPCR) analyses of the SDPP samples were used to infer the potential viral contamination in the liquid porcine plasma from which it was produced.

Material and Methods

Ethical statement

No animals were used for the study conducted.

Spray-dried porcine plasma sample collection

One sample per month was collected from a randomly selected commercial lot of SDPP during 12 consecutive months from eight different manufacturing plants located in Iowa, USA (IA-USA), North Carolina, USA (NC-USA), Santa Catarina, Brazil (SC-Brazil), central Spain (C-Spain), northeastern Spain (NE-Spain), central England (C-England) and Northern Ireland (N-Ireland). The N-Ireland manufacturing plant collect porcine blood from abattoirs located both, in Republic of Ireland and Northern Ireland. Samples from a manufacturing plant located in Quebec, Canada (QB-Canada), were taken biweekly during a 6 month-period.

Samples were collected from July 2018 to June 2019 (SC-Brazil), August 2018 to July 2019 (IA-USA, NE-Spain, C-Spain and N-Ireland) or September 2018 to August 2019

(NC-USA, C-England). The QB-Canada plant provided 12 samples randomly collected from March to August 2019. The collected SDPP samples represented a single point in time, not the entire month. Whole blood or plasma was stored at the abattoir in agitated tanks, transported to the spray drying facility in dedicated tankers and stored and blended with other plasma from different slaughterhouses in agitated silos before drying. In the manufacturing plants used in this study, a manufacturing lot of SDPP can range between 3,000 to 30,000 Kg of plasma depending on the plant. Therefore, one lot of SDPP represented between 16,650 to 166,500 pigs. During the 12-month collection period, samples were stored in whirl packs (Whirl-Pak®, Nasco, Madison, WI) and held at each plant in the quality assurance laboratory (room temperature) during the collection period. Subsequently, all SDPP samples from each manufacturing plant were sent to the IRTA-CReSA Animal Health Research Center in Barcelona, Spain, and stored until analyses for virus genome. One sample collected in December from the IA-USA plant was damaged during transport and was not used for analysis. Therefore, a total of 95 SDPP samples were analyzed.

Sample analysis by PCR

All SDPP samples were re-solubilized in distilled water at the ratio 1:9 of SDPP: water volume to represent the typical solid content in liquid plasma. Two hundred milliliters of diluted plasma sample were used for nucleic acid extraction using MagMAX™ Pathogen RNA/DNA Kit (Thermo Fisher Scientific, MA, USA). The recommended quantity of purified nucleic acids ~~were~~ amplified using real time PCR kits for PCV-2 (LSI VetMAX™ Porcine Circovirus Type 2 Quantification, Thermo Fisher Scientific, MA, USA), *Porcine reproductive and respiratory syndrome virus* [PRRSV] European and North American

strains (LSI VetMAX™ PRRSV EU/NA Real-Time PCR Kit; Thermo Fisher Scientific, MA, USA), *Swine influenza virus* [SIV] (EXOone Influenza A, EXOPOL, Zaragoza, Spain), *Porcine parvovirus* [PPV] (VetMAX™ Porcine Parvovirus Kit, Thermo Fisher Scientific, MA, USA), PEDV, *Transmissible gastroenteritis virus* [TGEV] and Swine deltacoronavirus [SDCoV] (VetMAX™ PEDV/TGEV/SDCoV, Thermo Fisher Scientific, MA, USA) and *Senecavirus A* [SVA] (EXOone Seneca Virus Valley, EXOPOL, Zaragoza, Spain).

According to all PCR kit guidelines, virus genome results with Ct values >40 were considered negative.

Virus stock production for development of standard curves to convert PCR Ct to $TCID_{50}$/g SDPP.

From those viruses detected in SDPP by qPCR, a stock of each virus was produced in the laboratory. Seven serial dilutions of viral stocks (PEDV, PRRSV-1 (EU strain), PRRSV-2 (US strain), PPV-1, PCV2 and SIV) were analyzed by quantitative PCR/RT-PCR (obtaining the corresponding Ct value) and $TCID_{50}$ titrated. Standard curves were established for each virus by regressing $TCID_{50}$/g SDPP on Ct results [Fig 1]. Those viral stocks were used as an internal standard on each amplification run/plate and quantitative PCR/RT-PCR Ct values extrapolated to $TCID_{50}$. Potential viral quantity determined on SDPP was corrected for typical solids content for each commercially collected plasma. $TCID_{50}$ titers were calculated by the Reed and Muench method [22].

Porcine reproductive and respiratory syndrome virus

Porcine reproductive and respiratory syndrome virus 3268 EU strain was propagated in porcine alveolar macrophages (PAM) grown in standard growth media (SGM) containing minimum essential medium eagle (MEM-E; ThermoFisher, Waltham, MA, USA) supplemented with 1% penicillin 10,000 U/mL and streptomycin 10 mg/mL (ThermoFisher), 0.5% Nystatin 10,000 IU/mL (Sigma-Aldrich, Burlington, MA, USA), 1% L-glutamine 200 mM (ThermoFisher) plus 5% fetal bovine serum (FBS). Cells were cultured in 75-cm^2 flasks. When cells were confluent, the media was discarded, and the adsorption was done using the virus at 0.01 MOI. After 1.5 hours at 37ºC, inoculum was removed, and 30 mL of medium were added. Titration was done in triplicate obtaining a final titer of $10^{5.48}$ $TCID_{50}$/mL.

Porcine reproductive and respiratory syndrome virus RV2332 US strain was propagated in MARC145 cells (ATCC No. CRL-12231) (kindly provided by Dr. Enric Mateu, *Universitat Autònoma de Barcelona*, Barcelona, Spain) using SGM supplemented with 10% FBS as explained above until a viral stock solution with a final titer of $10^{4.95}$ $TCID_{50}$/mL was obtained.

Porcine epidemic diarrhea virus

Porcine epidemic diarrhea virus CV777 strain [23], kindly provided by Dr. Hans Nauwynck (University of Ghent, Belgium), was propagated in VERO cells (ATCC CCL-81) grown in SGM with 10% FBS. Cells were cultured in 175-cm^2 flask and when they were confluent, the media was removed, and cells were rinsed twice with PBS. Finally, inoculum was added at 0.001 MOI and adsorption was done for 1 hour at 37ºC. Subsequently, the inoculum was discarded, flasks were rinsed twice with PBS and SGM supplemented with 10

mg/mL trypsin, and 0.3% tryptose (Sigma-Aldrich, Burlington, MA, USA). The viral stock was produced in the same cells and was titrated in triplicate obtaining a suspension with a viral titer of $10^{5.42}$ $TCID_{50}$ /mL.

Swine influenza virus

Swine influenza virus strain H1N1 A/Swine/Spain/SF11131/2017 [24] was propagated in MDCK cell line (ATCC CCL-34) grown in DMEM (ThermoFisher, Waltham, MA, USA) supplemented with 1% penicillin (10,000 U/mL), 1% streptomycin (10 mg/mL; ThermoFisher), 0.5% Nystatin (10,000 U/mL) (Sigma-Aldrich, Burlington, MA, USA), 1% L-glutamine 200mM (ThermoFisher) and 5% FBS. Cells were cultured in 175-cm^2 flask. When cells were confluent, the media was discarded, and the adsorption was done at 0.1 MOI. After 1 hour at 37ºC, inoculum was removed, and 30 mL of medium were added. The viral suspension was titrated in triplicate and the final virus titer was $10^{7.558}$ $TCID_{50}$ /mL.

Porcine circovirus 2

Porcine circovirus 2 genotype b isolate Sp-10-7-54-13 [25] was cultured in the PK-15 cell line (provided by the Institute of Virology UE and OIE Reference Laboratory for CSFV, Hannover), grown in SGM with 10% FBS. A mix of 6 mL of virus stock and 7 x 10^6 PK-15 cells resuspended in 50 mL of MEM-E (MOI 0.1) were added in 175 and 25 cm^2 flasks. At 24 hours cells were treated with glucosamine (Sigma-Aldrich, Burlington, MA, USA) to facilitate the virus infection. Forty-eight hours later, viral infection was checked by

IPMA [26] in the 25 cm^2 flask. If more than 25 positive cells were counted in a microscope field, the 175 cm^2 flask was trypsinized and the cells were transferred to 3 new 175 cm^2 flasks. The virus stock was titrated in triplicate with a final titer of $10^{5.5}$ $TCID_{50}$ /mL.

Porcine parvovirus

Porcine parvovirus strain NADL-2 was kindly provided by Dr Albert Bosch (Department of Genetics, Microbiology and Statistics School of Biology, University of Barcelona, Spain). It was propagated in SK-RST cells (ATCC CRL-2842), grown in SGM supplemented with 5% FBS. One mL of virus stock and 9 mL of MEM-E supplemented with 1% pyruvate (Merck KGaA, Darmstadt, Germany) were added to a conical tube with 16 x 10^6 SK-6 cells and shaken for 30 minutes at 104 rpm and 37ºC. After that time, the contents of the tube were transferred to a 175 cm^2 flask, in which 40 mL of MEM-E supplemented with 1% pyruvate were added. A viral suspension was obtained and titrated in triplicate, obtaining a final viral solution of $10^{6.64}$ $TCID_{50}$ /mL.

Estimation of $TCID_{50}$ and genomic equivalent copies (GEC) from Ct values obtained from q-PCR results.

To establish correspondence of positive qPCR results (measured as Ct values) with $TCID_{50}$/mL and viral genome copy content (GCC), seven serial dilutions of abovementioned titrated virus stocks were performed, and virus genome amplified with a second set of PCR kits (GPS, Genetic PCR Solutions Alicante, Spain). Each kit contained a

genome quantified standard for the different viruses tested: PRRSV (PRRSV-I dtec-RT-qPCR, PRRSV-II dtec-RT-qPCR), PEDV (PEDV dtec-RT-qPCR), PPV (PPV-1 dtec-RT-qPCR) and SIV (SIV dtec-RT-qPCR).

Statistical analysis

Dilutions of titrated viral stocks were included as an internal standard on each amplification PCR run containing SDPP samples. The Excel software was used to obtain the equation correlating $TCID_{50}$ and Ct values as well as GEC and Ct values. Then, results of the different PCR techniques originally expressed as Ct values for each SDPP sample tested were extrapolated to virus infectious particles and GEC based on the obtained regression formulae.

Average, number of observations, standard deviation, minimum value, maximum value, and ranges were calculated within each virus and for each SDPP producing plant using LSMEANS (SAS 9.4, 2016).

Results and Discussion

In this survey, viral loads for several porcine pathogens including SVA, TGEV, PRRSV (EU and US strains), PEDV, PCV2, SIV, SDCoV and PPV were determined by qPCR in reconstituted commercial SDPP. First, the Ct values from serial dilutions of a stock solution for each virus allowed the development of a correlation equation between Ct and $TCID_{50}$ that subsequently enabled estimating the putative viral titers in the SDPP

samples. Finally, using typical solids content of unprocessed liquid plasma, the viral level in liquid plasma was adjusted per gram ($TCID_{50}$/g liquid plasma). The relationships between Ct and $TCID_{50}$ of serial diluted stock solutions were linear with a correlation coefficient from 0.95 to 0.995 (Fig 1). Similar correlation coefficients were found when regressing Ct on $\log_{10}$ GEC/g on the tested samples (Fig 1). The slope of the lines for either $TCID_{50}$ or GEC/g were similar, while the intercepts were different (Fig 1), consistent with the fact that not all viral genome copies are infective [27]. In fact, high variability between infectious particles and genome copy numbers were observed among tested viruses, with less than 1 log difference for SIV to around 4 log differences for PCV-2.

Previous research has shown PCR/RT-PCR Ct values in SDPP to be relatively stable during normal storage conditions [19, 28, 29]. Similar levels of viral genome were detected in plasma inoculated with PCV-2 or SIV before and after spray drying (data not shown). The stability of PCR Ct values, the linear relationship between Ct and $TCID_{50}$ and the linear relationship between Ct and GEC provides additional assurance that estimated viral contamination of commercially collected SDPP and estimates of liquid plasma are accurate.

Frequency of detection and estimated quantity of virus in SDPP samples mimicking unprocessed liquid plasma samples collected at different plants is presented in Tables 2 and 3. S1 Table includes Ct values and estimated virus levels reported as Log_{10} GEC/g and Log_{10} $TCID_{50}$/g in reconstituted SDPP from the different manufacturing plants. S2 Table includes the estimated viral levels in unprocessed plasma reported Log$_{10}$ $TCID_{50}$/g. It is

important to recognize that a positive PCR/RT-PCR does not imply infectivity [16], fact that was observed for all the viruses studied in the present work.

In this survey neither SVA, TGEV nor SDCoV were detected in any of the SDPP samples (data not shown). SVA infection has been detected in the Americas and Asia, but not in Europe [30], so, it was expectable to have a significant number of RT-PCR negative samples. Importantly, viremia and clinical signs in SVA infected pigs appear within 2 to 3 days post-inoculation [31, 32], so, it is very difficult to have pigs in its highest shedding phase without displaying clinical signs. Despite SVA infected animals are sporadically detected on-farm and at abattoirs during ante-mortem inspection [33], effective identification of non-symptomatic animals probably contributed to the absence of SVA genome in the tested SDPP samples. Further supporting this hypothesis, a US survey reported only 1.2% of oral samples from 25 states being RT-PCR positive for SVA [34]. On the other hand, the inability to detect TGEV in these samples is also consistent with a very low incidence in the US and European swine population [35-37]. In case of SDCoV, the current data agree with prevalence results from Puente et al. [38] that indicated absence of SDCoV and TGEV in 106 Spanish pig farms analyzed between 2017-2019. Furthermore, Ajayi et al. [39] indicated that the presence of SDCoV in Ontario farms decrease from 1.14% in 2014 to 0.08% in 2016, matching with our results of very low presence of SDCoV in the North American pig population analyzed in 2018-19. Noteworthy, samples from Brazil were negative for both PRRSV and PEDV, which is consistent with other reports indicating that these viruses are not present in this country [40-45].

All SDPP samples were tested for both the EU and US strains of PRRSV independently of the geographical origin of the SDPP. Samples from the US contained PRRSV genotype 2, except for one sample from US-IA that had a PRRSV genotype 1 RT-PCR positive result (Ct of 35.70, equivalent to -0.29 Log_{10} $TCID_{50}$/g SDPP). Similarly, the samples from EU contained the PRRSV genotype 1, except for one sample from Spain-C that had PRRSV genotype 2 positivity (Ct of 36.08, equivalent to -2.09 Log_{10} $TCID_{50}$/g SDPP). The detection frequency of positive samples differed between plants, with 100% in those from US-IA, 17% in US-NC and 50% in Canada production plants. In Europe, the RT-PCR positivity against PRRSV was 33% for Spain-NE, 58% for Spain-C, 50% for England and 83% for N-Ireland. However, in both the US and in the EU, the estimated PRRSV $TCID_{50}$ in SDPP was very low (< 2 virus particle/g SDPP, average Ct of 33.83 ± 1.83 and 33.99 ± 1.26 for genotype 2 and 1, respectively in SDPP samples). Other works have reported low incidence of PRRSV viremia in slaughtered aged pigs [46] and differences in infection prevalence among US geographical areas [47], which ~~would be~~ aligned with the results obtained in the present survey.

Estimated PEDV levels in liquid plasma ~~was~~ low (<2.00 log_{10} PEDV/g SDPP). The detection frequency of positive samples was 82% in US-IA, 50% in US-NC and 8% in Canada. These results indicated that PEDV genome distribution was low in Eastern Canada compared with the USA and agrees with surveillance of PEDV cases reported in North America [48, 49]. In Europe, the incidence of positive PEDV samples was 83% in Spain-NE, and 67% in Spain-C while in England and N-Ireland the samples were negative. Although the present study was not designed to elucidate seasonal differences in the estimated quantity for PEDV genome in the different parts of the world, obtained results

suggest higher frequency of detection and viral loads during the winter, while it was lower in summertime (S1 and S2 Tables). These results are in line with the fact that PEDV is more stable in cold environments [50] and has lower incidence of clinical diarrhea cases at farms during the summer season [51].

Both PPV and PCV-2 are stable non-enveloped DNA viruses [52, 53]. Frequency of detection of both PPV and PCV-2 was 100%, since all samples tested positive for genetic material. In all regions, the estimated level of PCV2 was low (<2 $\log_{10}$ $TCID_{50}$/g SDPP that correspond to less than 5 virus particle per g of raw plasma), while PPV presence was slightly higher (<2.0 l[illegible] $TCID_{50}$/g liquid plasma). Other studies have reported low levels of PCV-2 viremia in finishing swine [54, 55], in part due to the widespread use of PCV-2 vaccine [56, 57]. In addition, PCV-2 natural infection mainly occurs during the nursery and growing periods, so, most of animals reach slaughterhouse immunized and with low levels or no virus circulating [58]. On the other hand, PPV vaccines are widely used in sows all over the world; considering the duration of PPV maternally derived immunity [53], it was expected to have evidence of natural infection in late finisher pigs. This aspect was confirmed with the present study.

Detection frequency of SIV RNA was very sporadic and the range of potential viral contamination was variable. In IA, NC and Canada, 9%, 0% and 8% of samples yielded positive results, respectively, and estimated amount of viable virus was <1.0 $\log_{10}$ $TCID_{50}$/g SDPP. Similarly, the frequency of detection of SIV in Spain-C, Spain-NE, England, N-Ireland and Brazil was 17%, 17%, 25%, 8% and 25%, respectively. However, when present, had a very wide range of viral loads from 0.32 to 5.59 Log_{10} $TCID_{50}$/g SDPP (corresponding to -0.72 to 4.56 Log_{10} $TCID_{50}$/g liquid raw plasma). It is speculated that

differences in stunning method, design of collection trough or slower line speed of abattoirs in Europe and Brazil compared to that in US and Canada may contribute to different levels of SIV contamination

Estimated levels of infectious viruses in commercially collected pork plasma was significantly lower than viral levels at peak viremia of pigs [31, 46, 56, 59]. Commercially collected porcine plasma is harvested from animals that have been inspected and passed as fit for slaughter for human consumption, precluding collection of blood from clinically sick animals. Typically, market hogs have been vaccinated for many of the economically important diseases and have developed effective immunity [60, 61]. Therefore, data from this survey suggest that potential viral contamination in commercially produced SDPP is very low. Theoretical combined inactivation for all viruses analyzed in this study of multiple ~~hurdles~~ can inactivate more than 6 $\log_{10}$ $TCID_{50}$/g SDPP for spray drying and post drying storage and > 10 $\log_{10}$ $TCID_{50}$/g SDPP if UV-C if also included (Table 1).

In summary, the data from this survey allowed the calculation of potential viral contamination in commercially collected porcine plasma. Estimated level of viral contamination in commercially collected porcine plasma was very low ranging from <2 $\log_{10}$ $TCID_{50}$ for most viruses with infrequent SIV levels as high as 4.5 $\log_{10}$ $TCID_{50}$/g liquid plasma. Considering that the multiple ~~hurdles~~ in the manufacturing process (UV-C, spray drying and post drying storage) are theoretically capable of inactivating much higher levels of virus (11 to 20 $\log_{10}$ $TCID_{50}$), it is safe to assume that SDPP is a product devoid of infectious virus particles. These data suggest that the multiple ~~hurdles~~ in the manufacturing process of SDPP should be sufficient to inactivate much higher loads of viruses than the

potential viral contamination that can be detected in commercially collected porcine plasma.

Acknowledgments

The authors want to appreciate the help provided by the manufacturing and quality assurance staff of all the manufacturing plants involved in this research for their support providing the samples used in this study.

Conflict of interest

The authors have read the journal's policy and the authors of this manuscript have the following competing interests: EB, CR, and JPolo are employed by APC Europe, S.L.U. Granollers, Spain and JC, LR and JPolo are employed by APC LLC, Ankeny, IA, USA. APC Europe and APC LLC manufactures and sells spray-dried animal plasma; however, the companies did not have any additional role in the study design, data collection and analysis, decision to publish, or preparation of the manuscript. This does not alter the authors' adherence to all PLOS ONE policies on sharing data and materials. JPujols, and JS declared no conflict of interest.

References

1. Torrallardona D. Spray dried animal plasma as an alternative to antibiotics in weanling pigs. Asian-Australasian J Anim Sci. 2010; 23: 131–48. https://doi.org/10.5713/ajas.2010.70630
2. Remus A, Andretta I, Kipper M, Lehnen CR, Klein CC, Lovatto PA, et al. A meta-analytical study about the relation of blood plasma addition in diets for piglets in the post-weaning and productive performance variables. Livest Sci, 2013; 155: 294–300. https://doi.org/10.1016/j.livsci.2013.04.020
3. Pérez-Bosque A, Polo J, Torrallardona D. Spray dried plasma as an alternative to antibiotic in piglet feeds, mode of action and biosafety. Porcine Health Manag. 2016; 2:16. doi:10.1186/s40813-016-0034-1.
4. Campbell JM, Crenshaw JD, Gonzalez-Esquerra R, Polo J. Impact of spray-dried plasma on intestinal health and broiler performance. Microorganisms. 2019; 7: 219. doi:10.3390/microorganisms7080219.
5. Patterson AR, Madson DM, Opriessnig T. Efficacy of experimentally produced spray-dried plasma on infectivity of porcine circovirus type 21J Anim Sci. 2010; 88:4078–4085. doi:10.2527/jas.2009-2696.
6. Pasick J, Berhane Y, Ojkic D, Maxie G, Embuty-Hyatt C, Swekla K, et al. Investigation into the role of potentially contaminated feed as a source of the first-detected outbreaks of porcine epidemic diarrhea in Canada. Transbound Emerg Dis. 2014; 61: 397–410. https://doi.org/10.1111/tbed.12269
7. Aubry P, Thompson JL, Pasma T, Furness MC, Tataryn J. Weight of the evidence linking feed to an outbreak of porcine epidemic diarrhea in Canadian swine herds. J Swine Health & Prod. 2017; 25(2): 69–72. https://www.asi.k-state.edu/research-and-

extension/swine/Compressed%20Feed%20linked%20to%20PEDV%20outbreak%20in%20Canada.pdf

8. Messier S, Gagne-Fortin C, Crenshaw J. Dietary spray-dried porcine plasma reduces mortality attributed to porcine circovirus associated disease syndrome. Proc. Amer. Assoc. Swine Vet. 2007; p 147-150.
9. Pujols J, Segalés J, Polo J, Rodríguez C, Campbell J, Crenshaw J. Influence of spray dried porcine plasma in starter diets associated with a conventional vaccination program on wean to finish performance. Porcine Health Manag. 2016; 2:4. doi 10.1186/s40813-016-0021-6.
10. Dewey CE, Johnston WT, Gould L, Whiting TL. Postweaning mortality in Manitoba swine. Can J Vet Res. 2006; 70:161–167. PMID: 16850937; PMCID: PMC1477929.
11. Shen HG, Schalk S, Halbur PG, Campbell JM, Russell LE, Opriessnig T. Commercially produced spray-dried porcine plasma contains increased concentrations of porcine circovirus type 2 DNA but does not transmit porcine circovirus type 2 when fed to naive pigs. J Anim Sci. 2011; 89:1930–1938. doi:10.2527/jas.2010-3502.
12. Russell LE, Polo J, Meeker D. 2020. The Canadian 2014 porcine epidemic diarrhoea virus outbreak: Important risk factors that were not considered in the epidemiological investigation could change the conclusions. Transbound Emerg Dis. 2020;67:1101–1112. doi: 10.1111/tbed.13496.
13. Polo J, Quigley JD, Russell LE, Campbell JM, Pujols J, Lukert PD. Efficacy of spray-drying to reduce infectivity of pseudorabies and porcine reproductive and respiratory syndrome (PRRS) viruses and seroconversion in pigs fed diets

containing spray-dried animal plasma. J Anim Sci. 2005; 83: 1933–1938. https//doi.org/10.2527/2005.8381933x

14. Pujols J, Rosell R, Russell L, Campbell J, Crenshaw J. Inactivation of swine vesicular disease virus in porcine plasma by spray-drying. Am Assoc Swine Vet. 2007; Perry, IA: p.281-284.
15. Gerber PF, Xiao C-T, Chen Q, Zhang J, Halbur PG, Opriessnig T. The spray-drying process is sufficient to inactivate infectious porcine epidemic diarrhea virus in plasma. Vet. Microbiol. 2014; 7;174(1-2):86-92. doi:10.1016/j.vetmic.2014.09.008.
16. Pujols J, Segalés J. Survivability of porcine epidemic diarrhea virus (PEDV) in bovine plasma submitted to spray drying processing and held at different time by temperature storage conditions. Vet Microbiol. 2014; 174: 427–432. doi: 10.1016/j.vetmic.2014.10.021.
17. Blázquez E, Rodríguez C, Ródenas J, Navarro N, Riquelme C, Rosell R, et al. Evaluation of the effectiveness of the SurePure Turbulator ultraviolet-C irradiation equipment on inactivation of different enveloped and non-enveloped viruses inoculated in commercially collected liquid animal plasma. PLoS One. 2019;14:e0212332. https://doi.org/10.1371/journal.pone.0212332.
18. Blázquez E, Rodríguez C, Ródenas J, Segalés J, Pujols J, Polo J. Biosafety steps in the manufacturing process of spray-dried plasma: a review with emphasis on the use of ultraviolet irradiation as a redundant biosafety procedure. Porcine Health Manag. 2020; 6:16. https://doi.org/10.1186/s40813-020-00155-1.
19. Fischer M, Pikalo J, Beer M, Blome S. Stability of African swine fever virus on contaminated spray dried porcine plasma. Transbound Emerg Dis. 2021;1-6. doi: 10.1111/tbed.14192.

20. WHO. Annex 4 Guidelines on viral inactivation and removal procedures intended to assure the viral safety of human blood plasma products, vol.924: Geneva: World Health Organisation; 2004. p. 150–224.

21. Goodrich RP, Custer B, Keil S, Busch M. Defining "adequate" pathogen reduction performance for transfused blood components. Transfusion 2010; 50:1827-1837. doi: 10.1111/j.1537-2995.2010.02635.x

22. Reed MJ, Muench H. A simple method for estimating fifty percent end points. Am J Hyg. 1938; 27: 493-497. https://doi.org/10.1093/oxfordjournals.aje.a118408

23. Debouck P, Pensaert M. Experimental infection of pigs with a new porcine enteric coronavirus, CV 777. Am. J. Vet. Res. 1980; 41: 219–223. PMID: 6245603.

24. Baratelli M, Córdoba L, Pérez LJ, Maldonado J, Fraile L, Núñez JL, et al. Genetic characterization of influenza A viruses circulating in pigs and isolated in north-east Spain during the period 2006-2007. Res Vet Sci. 2014; 96: 380-388. doi: 10.1016/j.rvsc.2013.12.006.

25. Fort M, Sibila M, Nofrarías M, Pérez-Martín E, Olvera A, Mateu E, et al. Porcine circovirus type 2 (PCV2) Cap and Rep proteins are involved in the development of cell-mediated immunity upon PCV2 infection. Vet Immunol Immunopathol. 2010; 137: 226–234. doi: 10.1016/j.vetimm.2010.05.013.

26. Rodríguez-Arrioja GM, Segalés J, Calsamiglia M, Resendes AR, Balasch M, Plana-Duran J, et al. Dynamics of porcine circovirus type 2 infection in a herd of pigs with postweaning multisystemic wasting syndrome. Am J Vet Res. 2002; 63: 354–357. doi: 10.2460/ajvr.2002.63.354.

27. Parker J, Fowler N, Walmsley M-L, Schmidt T, Scharrer J, Kowaleski J, et al. Analytical sensitivity comparison between singleplex real-time PCR and a

multiplex PCR platform for detecting respiratory viruses. Plos One. 2015; 10(11):e0143164. doi: 10.1371/journal.pone.0143164.

28. Cochrane RA, Dritz SS, Woodworth JC, Huss AR, Stark CR, Hesse RA, et al. Evaluating Chemical Mitigation of Porcine Epidemic Diarrhea Virus (PEDV) in Swine Feed and Ingredients. Kansas Agricultural Experiment Station Research Reports 2015; Vol. 1: Iss. 7. http://dx.doi.org/10.4148/2378-5977.1110.

29. Dee S, Neill C, Clement T, Singrey A, Christopher-Hennings J, Nelson E. An evaluation of porcine epidemic diarrhea virus survival in individual feed ingredients in the presence or absence of a liquid antimicrobial. Porc Health Manag 2015; 1: 9. https://doi.org/10.1186/s40813-015-0003-0.

30. Houston E, Temeeyasen G, Piñeyro PE. Comprehensive review on immunopathogenesis, diagnostic and epidemiology of Senecavirus A. Virus Res. 2020; 286:198038. doi: 10.1016/j.virusres.2020.198038.

31. Joshi LR, Fernandes MHV, Clement T, Lawson S, Pillatzki A, Resende TP, et al. Pathogenesis of Senecavirus A infection in finishing pigs. J Gen Virol. 2016; 97: 3267–3279. doi: 10.1099/jgv.0.000631.

32. Zhang H, Chen P, Hao G, Liu W, Chen H, Qian P, et al. Comparison of the Pathogenicity of Two Different Branches of Senecavirus a Strain in China January 2020.Pathogens 2020; 9(1): 39. doi:10.3390/pathogens9010039.

33. Baker KL, Mowrer C, Canon A, Linhares DCL, Rademacher C, Karriker LA, et al. Systematic Epidemiological Investigations of Cases of Senecavirus A in US Swine Breeding Herds. Transbound Emerg Dis. 2017; 64:11–18. doi:10.1111/tbed.12598.

34. Leme RA, Alfieri AF, Alfieri AA. Update on Senecavirus Infection in Pigs. Viruses. 2017; 9:170. doi:10.3390/v9070170.

35. Schwegmann-Wessels C, Herrler G. Transmissible gastroenteritis virus infection: a vanishing specter. Dtsch Tierarztl Wochenschr. 2006; 113(4):157-159. PMID: 16716052.

36. Pensaert MB, Martelli P. Porcine epidemic diarrhea: a retrospect from Europe and matters of debate. Virus. Res. 2016; 226:1-6. doi: 10.1016/j.virusres.2016.05.030.

37. Chen F, Knutson TP, Rossow S, Saif LJ, Marthaler DG. Decline of transmissible gastroenteritis virus and its complex evolutionary relationship with porcine respiratory coronavirus in the United States. Scient Rep. 2019; 9:3953. https://doi.org/10.1038/s41598-019-40564-z.

38. Puente H, Argüello H, Mencía-Ares O, Gómez-García M, Rubio P, Carvajal A. Detection and genetic diversity of porcine cornavirus involved in diarrhea outbreaks in Spain. Front. Vet. Sci. 2021; 8: 651999. doi: 10.3389/fvets.2021.651999.

39. Ajayi T, Dara R, Misener M, Pasma T, Moser L, Poljak Z. Herd-level prevalence and incidence of porcine epidemic diarrhoea virus (PEDV) and porcine deltacoronavirus (PDCoV) in swine herds in Ontario, Canada. Transbound Emerg Dis. 2018; 65:1197-1207. doi: 10.1111/tbed.12858.

40. Crenshaw JD, Campbell JM, Polo J. Analysis of spray dried porcine plasma (SDPP) produced in Brazil and Western Canada confirm negative porcine epidemic diarrhea virus (PEDv) status of pigs in these regions. Proc. Allen D. Leman Swine Conf. 2014. Recent Research Reports, Univ. MN, St. Paul, MN. Sept. 13-16, 40:14.

41. Crenshaw J, Pujols J, Polo J, Campbell J, Rodríguez C, Navarro N, et al. Analysis of spray dried porcine plasma indicates absence of PRRSV infection in Brazilian pigs. 23rd IPVS Congress 2014. Cancun, México. June 8-11, 2014. p. 556. Poster 576.

42. Gava D, Caron L, Schaefer R, Santiago-Silva V, Weiblen R, Furtado-Flores E, et al. A retrospective study of porcine reproductive and respiratory syndrome virus infection in Brazilian pigs from 2008 to 2020. Transbound Emerg Dis. 2021; 00:1–5. doi: 10.1111/tbed.14036.

43. OIE, World Organisation of Animal Health. World Animal Health Information Database (WAHIS) Interface. Available from: https://www.oie.int/wahis_2/public/wahid.php/Diseaseinformation/statuslist.

44. Rech RR, Gava D, Silva MC, Fernandes LT, Haach V, Ciacci-Zanbella JR, et al. Porcine respiratory disease complex after the introduction of H1N1/2009 influenza virus in Brazil. Zoonoses Public Health. 2018; 65(1):e155-e161. doi:10.1111/zph.12424.

45. Zanella JRC, Morés N, de Barcellos DESN. Principais ameaças sanitárias endêmicas da cadeia produtiva de suínos no Brasil. Pesq. agropec. bras., Brasília. 2016; 51 (5):443-453. doi: 10.1590/S0100-204X2016000500004.

46. Lyoo KS, Choi JY, Hahn TW, Park KT, Kim HK. Effect of vaccination with a modified live porcine reproductive and respiratory syndrome virus vaccine on growth performance in fattening pigs under field conditions. J Vet Med Sci. 2016; 78(9):1533-1536. doi: 10.1292/jvms.16-0137.

47. Alkhamis MA, Arruda AG, Vilalta C, Morrison RB, Perez AM. Surveillance of porcine reproductive and respiratory syndrome virus in the United States using risk mapping and species distribution modeling. Prev Vet Med. 2018;150, 135–142. https://doi.org/10.1016/J.PREVETMED.2017.11.011

48. CSHIN quarterly producer report. Can Swine Health Intelligent Network. 2019. Available from: https://www.cpc-

ccp.com/uploads/userfiles/files/CSHIN%202019%20Q3%20Producer%20Report_FINAL%20EN.pdf

49. Machado G, Vilalta C, Recamonde-Mendoza M, Corzo C, Torremorell M, Pérez A, et al. Identifying outbreaks of porcine epidemic diarrhea virus through animal movements and spatial neighborhoods. Scient Rep. 2019; 9:457. doi: 10.1038/s41598-018-36934-8.
50. Saif L, Pensaert M, Sestak K, Yeo S, Jung K. Coronaviruses. In, Zimmerman,J., Karriker,L., Ramirez,A., Schwartz,K., and Stevenson,G. (eds), Diseases of Swine. John Wiley & Sons, Inc., Hoboken, NJ, USA, 2012:501–524.
51. Kong F, Xu Y, Ran W, Yin B, Feng L, Sun D - Cold Exposure-Induced Up-Regulation of Hsp70 Positively Regulates PEDV mRNA Synthesis and Protein Expression In Vitro. Pathogens. 2020; 9(4): 246. https://doi.org/10.3390/pathogens9040246
52. Segalés J, Allan GM, Domingo M. Circoviruses. In: Zimmerman JJ, Karriker LA, Ramirez A, Schwartz KJ, Stevenson GW, Zhang J, editors. Diseases of swine. 11th ed., Hoboken: John Wiley & Sons, Inc. 2019; 473-487. doi:10.1002/9781119350927.
53. Truyen U, Streck AF. Parvoviruses. In: Zimmerman JJ, Karriker LA, Ramirez A, Schwartz KJ, Stevenson GW, Zhang J, editors. Diseases of swine. 11th ed., Hoboken: John Wiley & Sons, Inc. 2019: 611–621. doi:10.1002/9781119350927.
54. Dvorak CMT, Yang Y, Haley C, Sharma N, Murtaugh MP. National reduction in porcine circovirus type 2 prevalence following introduction of vaccination. Vet Microb. 2016; 189: 86-90. doi: 10.1016/j.vetmic.2016.05.002.

55. Oliver-Ferrando S, Segalés J, López-Soria S, Callén A, Merdy O, Joisel F, et al. Evaluation of natural porcine circovirus type 2 (PCV2) subclinical infection and seroconversion dynamics in piglets vaccinated at different ages. M Vet Res. 2016; 47(1):121. https://doi.org/10.1186/s13567-016-0405-2

56. Opriessnig T, Gerber PF, Xiao C-T, Halbur PG, Matzinger SR, Meng X-J. Commercial PCV2a-based vaccines are effective in protecting naturally PCV2b-infected finisher pigs against experimental challenge with a 2012 mutant PCV2. Vaccine. 2014; 32(34):4342-4348. doi: 10.1016/j.vaccine.2014.06.004.

57. Witvliet M, Holtslag H, Nell T, Segers R, Fachinger V. Efficacy and safety of a combined porcine circovirus and Mycoplasma hyopneumoniae vaccine in finishing pigs. Trials Vaccinol. 2015; 4:43-49. http://dx.doi.org/10.1016/j.trivac.2015.04.002.

58. Rose N, Opriessnig T, Grasland B, Jestin A. Epidemiology and transmission of porcine circovirus type 2 (PCV2). Virus Res. 2012; 164(1-2):78-89. doi: 10.1016/j.virusres.2011.12.002.

59. Seo HW, Oh Y, Han K, Park C, Chae C. Reduction of porcine circovirus type 2 (PCV2) viremia by a reformulated inactivated chimeric PCV1-2 vaccine-induced humoral and cellular immunity after experimental PCV2 challenge. BMC Vet Res. 2012; 8:194. https://doi.org/10.1186/1746-6148-8-194

60. PIC Gilt and Sow Management Guidelines. 2021. PIC-Gilt-Sow-Management-Guidelines_05122%20(1).pdf

61. PIC Wean to Finish Guidelines. 2019. Wean-to-Finish-Manual-2019-Final%20(1).pdf

62. Sampedro F, Snider T, Bueno I, Bergeron J, Urriola PE, Davies PR. Risk assessment of feed ingredients of porcine origin as vehicles for transmission of Porcine Epidemic Diarrhea Virus (PEDv). National Pork Board. 2015;1–117.

63. Blázquez E, Rodríguez C, Ródenas J, Rosell R, Segalés J, Pujols J, et al. Effect of spray-drying and ultraviolet C radiation as biosafety steps for CSFV and ASFV inactivation in porcine plasma. Plos One. 2021; 16(4): e0249935. https://doi.org/10.1371/journal.pone.0249935

Table 1: Different inactivation steps involved in the manufacturing process of ~~SDPP~~. Inactivation expressed as Log_{10} $TCID_{50}$/g for viruses

Virus Type			Spray-Drying	UV-C*	Storage at 20ºC for 14 d	Combined Theoretical Inactivation	References
RNA	Enveloped	Porcine reproductive and respiratory syndrome virus (PRRSV)	>4.0	12.9 ± 0.3	>4.0	>20.9	[13, 17, 62]
		Swine influenza virus (SIV)	2.8**	7.9 ± 0.2	3.2**	13.9	[17]
		Porcine epidemic diarrhea virus (PEDV)	5.1<br>4.2	6.6 ± 0.1	3.8	14.6-15.5	[15-17]
		Classical swine fever virus (CSFV)	5.8	7.9 ± 0.2	~~ND~~	>13.7	[17, 63]
	Naked	Swine vesicular disease virus (SVDV)	6.7	3.5 ± 0.07	~~ND~~	>10.2	[14, 17]
		Senecavirus A (SVA)	ND	4.0 ± 0.08	>5.0**	>9.0	[17]
DNA	Enveloped	Pseudorabies virus (PRV)	5.3	8.1 ± 0.2	~~ND~~	>13.4	[13, 17]
		African swine fever virus (ASFV)	4.1 ± 0.2	6.8 ± 0.1	>5.0	>15.9	[17, 19, 63]
	Naked	Porcine parvovirus (PPV)	2.7**	6.0 ± 0.1	3.1**	>11.8	[17]

Values with > results indicate the inactivated amount in the processed sample exceeded the amount inoculated in the initial sample before processing or storage.

*The UV log-kill estimated values were calculated commercial UV dosage (3251 J/L) by the estimated D-value from Blázquez et al., [17].

**University of Minnesota. Understanding the risk of virus transmission in spray dried porcine plasma – food safety assessment. 2020. Unpublished data

Table 2. Ct values and estimated viral genome presence expressed in Log_{10} genome equivalent copies (GEC) and Log_{10} $TCID_{50}$/g ~~SDPP~~ in manufacturing plants located in different swine production areas around the world during the years 2018-2019. Values expressed as Average ± SD for ~~only~~ positive samples.

Plant	US-IA (n=11)	US-NC (n=12)	Canada (n=12)	Spain-NE (n=12)	Spain-C (n=12)	England (n=12)	NI (n=12)	Brazil (n=12)
PEDV								
Ct	33.46 ± 3.30	34.21 ± 2.06	33.94	35.09 ± 1.21	35.28 ± 1.39	Neg	Neg	Neg
Log_{10} GEC/g	2.87 ± 0.89	2.67 ± 0.56	2.74	2.43 ± 0.33	2.38 ± 0.38			
Log_{10} $TCID_{50}$/g	0.30 ± 0.92	0.10 ± 0.57	0.32	0.01 ± 0.33	-0.05 ± 0.38			
% Positive samples	82	50	8	83	67	0	0	0
PCV2								
Ct	31.68 ± 0.56	30.98 ± 1.72	29.83 ± 0.91	30.39 ± 0.64	30.17 ± 0.96	30.63 ± 1.24	30.80 ± 0.67	31.02 ± 0.37
Log_{10} GEC/g	5.29 ± 0.16	5.49 ± 0.50	5.67 ± 0.28	5.50 ± 0.20	5.57 ± 0.29	5.43 ± 0.38	5.38 ± 0.20	5.31 ± 0.11
Log_{10} $TCID_{50}$/g	1.40 ± 0.15	1.59 ± 0.47	1.76 ± 0.26	1.60 ± 0.18	1.66 ± 0.27	1.53 ± 0.35	1.48 ± 0.19	1.42 ± 0.11
% Positive samples	100	100	100	100	100	100	100	100
PPV								
Ct	30.44 ± 1.08	32.29 ± 2.44	30.64 ± 1.44	30.92 ± 3.25	30.58 ± 1.34	30.20 ± 1.08	28.42 ± 0.52	31.31 ± 1.02
Log_{10} GEC/g	3.96 ± 0.25	3.53 ± 0.57	3.92 ± 0.34	3.85 ± 0.77	3.93 ± 0.32	4.02 ± 0.25	4.44 ± 0.12	3.76 ± 0.24
Log_{10} $TCID_{50}$/g	2.81 ± 0.27	2.35 ± 0.61	2.79 ± 0.36	2.72 ± 0.81	2.81 ± 0.33	2.90 ± 0.27	3.34 ± 0.13	2.62 ± 0.25
% Positive samples	100	100	100	100	100	100	100	100
SIV								
Ct	37.55	Neg	35.42	23.17 ± 3.83	19.56 ± 0.28	23.62 ± 10.58	20.93	27.60 ± 9.53
Log_{10} GEC/g								
Log_{10} $TCID_{50}$/g	-1.33		0.44	3.93 ± 1.09	4.96 ± 0.08	3.80 ± 3.02	4.57	2.67 ± 2.72
% Positive samples	9	0	8	17	17	25	8	25
PRRS-US								
Ct	33.36 ± 1.64	34.29 ± 1.26	34.14 ± 2.24	Neg	36.08	Neg	Neg	Neg
Log_{10} GEC/g	2.40 ± 0.48	2.13 ± 0.37	2.17 ± 0.66		1.62			
Log_{10} $TCID_{50}$/g	-1.25 ± 0.51	-1.52 ± 0.39	-1.49 ± 0.69		-2.09			
% Positive samples	100	17	50	0	8	0	0	0
PRRS-EU								
Ct	35.70	Neg	Neg	34.51 ± 0.88	33.88 ± 1.60	33.64 ± 1.44	33.90 ± 1.04	Neg

Log_{10} GEC/g	2.08			2.42 ± 0.25	2.60 ± 0.46	2.67 ± 0.41	2.59 ± 0.30	
Log_{10} $TCID_{50}$/g	-0.29			0.03 ± 0.24	0.20 ± 0.43	0.26 ± 0.39	0.19 ± 0.28	
% Positive samples	9	0	0	33	58	50	83	0

Table 3. Estimated quantification of different viruses' genomes expressed in Log_{10} $TCID_{50}/g$ ± SD (percentage of positive samples) in unprocessed raw liquid plasma from PCR or RT-PCR analyses of ~~SDPP~~ samples collected at different plants.

Plant	**PEDV**	**PCV2**	**PPV**	**SIV**	**PRRS- US**	**PRRS-EU**
US-IA	-0.82 ± 0.92	0.28 ± 0.15	1.69 ± 0.27	-2.45	-2.37 ± 0.51	-1.42
US-NC	-0.94 ± 0.57	0.55 ± 0.47	1.31 ± 0.61	Neg	2.58 ± 0.39	Neg
Canada	-0.80	0.64 ± 0.26	1.67 ± 0.36	-0.69	-2.48 ± 0.65	Neg
Spain-NE	-1.03 ± 0.31	0.56 ± 0.18	1.68 ± 0.81	2.89 ± 1.09	Neg	-1.03 ± 0.25
Spain-C	-1.17 ± 0.38	0.54 ± 0.27	1.68 ± 0.33	3.84 ± 0.08	-3.21	-0.93 ± 0.43
England	Neg	0.49 ± 0.35	1.86 ± 0.27	2.76 ± 3.02	Neg	-0.78 ± 0.39
Northern Ireland	Neg	0.36 ± 0.19	2.22 ± 0.13	3.45	Neg	-0.93 ± 0.28
Brazil	Neg	0.30 ± 0.11	1.50 ± 0.25	1.56 ± 2.70	Neg	Neg
Range	-1.76 – -0.57	-0.28 – 1.37	-0.15 – 2.55	-2.45 – 4.56	-3.23 – [illegible]52	-1.47 – -0.18

Figure 1. Regression curves between Ct values and tissue culture infections dose 50 ($TCID_{50}$/g) or Genome equivalent copies (GEC)/g of spray-dried porcine plasma (SDPP).

Values expressed in Log_{10} $TCID_{50}$/g SDPP or Log_{10} GEC/g SDPP. Each box includes the spot values of the SDPP samples analyzed and the regression equation between CT and $TCID_{50}$/g or GEC/g SDP and the r^2 value.

A.Regression curves for *porcine epidemic diarrhea virus* (PEDV); B. Regression curves for *porcine circovirus type-2* (PCV2); C. Regression curves for *porcine parvovirus* (PPV); D. Regression curves for *swine influenza virus* (SVI) H1N1; E. Regression curves for *porcine reproductive and respiratory syndrome virus* (PRRSV) US strain; F. Regression curves for PRRSV EU strain.

Figure
Click here to access/download;Figure;Fig. 1. Comparative titres TCID50 vs Genome Copies -final.pptx

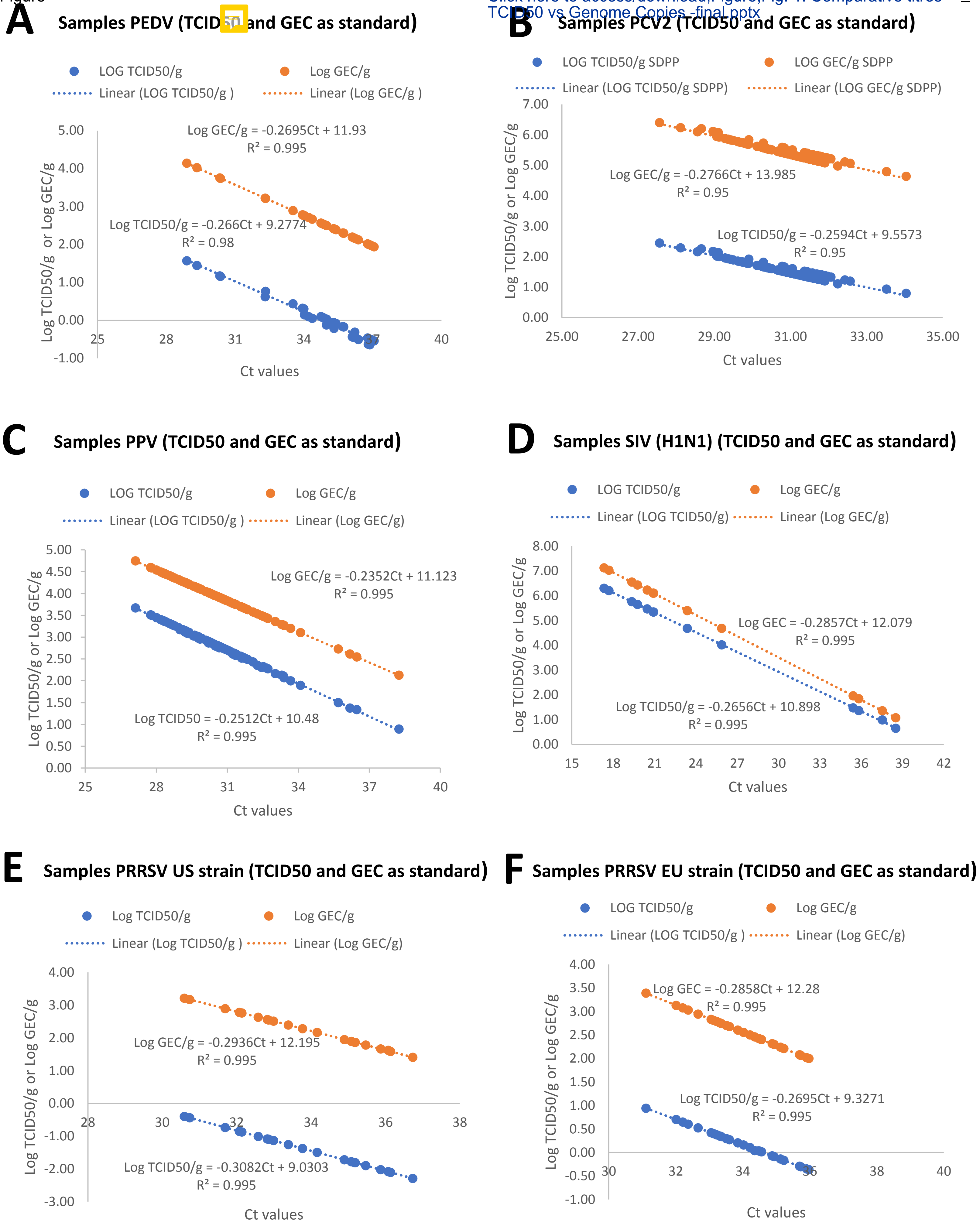

Click here to access/download
Supporting Information
Supplementary tables 1 & 2.xlsx
