# Supplementary material for: Estimated quantity of swine virus genomes based on quantitative PCR analysis in spray-dried porcine plasma samples collected from multiple manufacturing plants
Source: PLoS One. 2022 May 23;17(5):e0259613. doi: 10.1371/journal.pone.0259613 (PMC9126402; doi:10.1371/journal.pone.0259613)
Supplement: Attachment — Submitted filename: Response to reviewers-R1bis.docx [file pone.0259613.s004.docx]

2. We note that the grant information you provided in the ‘Funding Information’ and ‘Financial Disclosure’ sections do not match.

When you resubmit, please ensure that you provide the correct grant numbers for the awards you received for your study in the ‘Funding Information’ section.

Sorry we did not know how to include the funding information in the system. We add this information in the revised version of the manuscript

3. We note that you have included the phrase “data not shown” in your manuscript. Unfortunately, this does not meet our data sharing requirements. PLOS does not permit references to inaccessible data. We require that authors provide all relevant data within the paper, Supporting Information files, or in an acceptable, public repository. Please add a citation to support this phrase or upload the data that corresponds with these findings to a stable repository (such as Figshare or Dryad) and provide and URLs, DOIs, or accession numbers that may be used to access these data. Or, if the data are not a core part of the research being presented in your study, we ask that you remove the phrase that refers to these data.

We apologized for the misunderstanding. In fact, we provide the data of all the information included in the manuscript. We clarified this subject in the revised version.

4. We note you have included a table to which you do not refer in the text of your manuscript. Please ensure that you refer to Table 3 in your text; if accepted, production will need this reference to link the reader to the Table.

The Table 3 was already included in the text of the initial submission. Lines 234-235 as Tables 2 and 3. In the new revised version appears in line 236-237 as Table 2 and Table3.

5. Please include captions for your Supporting Information files at the end of your manuscript, and update any in-text citations to match accordingly. Please see our Supporting Information guidelines for more information: <http://journals.plos.org/plosone/s/supporting-information>.

Captions have been included for the Supporting information files. We have identified the supplementary tables as:

S1 Table -SDPP. Ct values and estimated virus genome presence in SDPP per months during the years 2018-2019.

S2 Table – Raw Plasma. Estimated virus genome presence in raw plasma per months during the years 2018-2019.

**Reviewer’s Comments to the Author**
**Reviewer #1:** This manuscript provides valuable information on the presence of genomic information for porcine viruses of concern in spray dried porcine plasma. The estimation of TCID50 based on standard curves is also of interest, as is the % positive sample information for SDPP from different geographical regions.

The authors appreciated the comments from Reviewer #1 because confirms our believe that the information provided in the manuscript can be of relevance for the swine sector, not only for understanding the presence of swine viral genome in raw or SDPP but also as a picture of the presence of different viruses in the different geographical areas studied. This information can be of importance for surveillance studies because provide quantitative information about the incidence of the different virus of concern for the swine industry.

I have annotated the manuscript with suggestions for improving English language usage and reader understanding. Please use these suggestions if you agree. I have also asked some questions that can be addressed in your revision.

The authors appreciated all the grammar suggestions provided by the Reviewer #1. We incorporated all of them in the revised manuscript.

Regarding the questions from reviewers #1. Find below the authors response to these questions.

Line # 55. “…multiple hurdles that…”. The reviewer is suggesting to change the word “hurdles” by “steps” throughout the manuscript.

To the authors, the term “multiple hurdles” is a common term to describe food manufacturing systems as can be found in the publication of Leistner (2000). Int. J. Food Microb. 55:181-186. <https://edisciplinas.usp.br/pluginfile.php/128773/mod_resource/content/1/Leistner.2000.pdf>

Therefore, since this is a usual wording for the field being here studied, the authors prefer to keep the term “multiple hurdles” throughout the article.

Line #56. “*…SD, 80oC throughout substance…”.* How much time?

Throughout substance means that the particle achieves 80ºC during the drying process but the time that the particle is at that temperature is unknown and is variable depending on the driers design and capacity. The safety heat treatment is to achieve this 80ºC. It is the same safety concept that in case of meat for human consumption, the heat treatment at a minimum temperature of 80°C, which must be reached throughout the meat but without specifying the time at that temperature (European Council Directive 2002/99/EC. Annex III).

Line #56. “…*ultraviolet light (UV) treatment (3000 J/L)…*”. J/m^2^

No, the values are correctly expressed as J/L and not J/m^2^ as suggested by the reviewer #1. We refer to the paper of Blázquez et al., 2019. Reference [17] in the manuscript. In the case of treating liquid plasma, the UV system is a continuous flow reactor designed for exposing a liquid to UV light. Therefore, J/L is the appropriate unit of measure.

Line #139. “…*After 1.5 hours at 37ºC, inoculum was removed, and 30 mL of medium were added. Titration was done in triplicate obtaining a final titer of 10^5.48^ TCID_50_/mL*.”. Two significant figures is enough. The titration assay is not accurate to three significant figures, I think.

The authors did not fully understand the comment from the reviewer #1. If he/she is referring that expressing the virus titer with maximum two decimals and not three as appear in some cases, we corrected this mistake in the revised version of the manuscript.

We assume that some were expressed with three significant figures due to the effect of the mathematical mean. We apologize for the mistake and thank you for correcting it.

Line #186. *“…A viral suspension was obtained and titrated in triplicate, obtaining a final viral solution of 10^6.64^ TCID_50_ /mL.* “ Provide incubation duration, is CPE the endpoint?

Sorry, we forgot to provide this information. Thank you for your observation. Once the flasks were inoculated, they were incubated at 37ºC for four days, at which time a clear CPE is observed and the infection was stopped.

We add this information on line 188 of the revised manuscript:

“*After that time, the contents of the tube were transferred to a 175 cm2 flask, in which 40 mL of MEM-E supplemented with 1% pyruvate were added****. Inoculated flasks were incubated for four days at 37ºC until CPE was observed.*** *A viral suspension was obtained and titrated in triplicate, obtaining a final viral solution of 10^6.64^ TCID_50_ /mL.* “

Line #197. *“… Each kit contained a genome quantified standard for the different vi 195 ruses tested: PRRSV (PRRSV-I dtec-RT-qPCR, PRRSV-II dtec-RT-qPCR), PEDV (PEDV dtec-RT-qPCR), PPV (PPV-1 dtec-RT-qPCR) and SIV (SIV dtec-RT-qPCR).*” Add information for PCV?

We do not provide information about PCV-2 in this section since in the case of PCV-2 it was not necessary to perform a quantitative PCR again due that the first PCR performed (PCV-2 (LSI VetMAXTM Porcine Circovirus Type 2 Quantification, Thermo Fisher Scientific, MA, USA)) was already quantitative.

Line #247. *“…effective identification of non-symptomatic animals probably contributed to the absence of SVA genome in the tested SDPP samples.*”. How are non-symptomatic animals identified?

SVA has been associated with lameness and cutaneous vesicles, as well as increased mortality in the first weeks of age. Therefore, the absence of these clinical signs, at least the ones affecting finishers would be one of the arguments by which SVA might not be detected. On the other hand, animals at abattoir are inspected ante-mortem, and presumably only animals non-displaying clinical signs are sacrificed and used for human consumption.

The reviewer is right that non-symptomatic animals is difficult to identified. We changed this paragraph in the revised version of the manuscript as following

“*Despite SVA infected animals have been sporadically detected on-farm and at abattoirs during ante-mortem inspection [33], effective identification of farm outbreaks and surveillance system in place probably contributed to the absence of SVA genome in the tested SDPP samples*”

Line #291. *“…In all regions, the estimated level of PCV2- was low (<2 log10 TCID50/g SDPP that corresponds to less than 5 virus particles per g of raw plasma), while PPV presence was slightly higher (<2.0 log10 TCID50/g liquid plasma)*.” This value is the same as that for PCV-2. Am I missing something?

The value for PCV-2 was indicated per g of SDPP while the value for PPV was refer to liquid plasma. In the revised version we put both values as g of SDPP. This sentence has been changed in the revised version of the manuscript as following:

“*In all regions, the estimated level of PCV-2 was <2 log_10_ TCID_50_/g SDPP, while PPV presence was <3.0 log_10_ TCID_50_/g SDPP*.”

Line #308. *“…It is speculated that differences in stunning method, design of collection trough or slower line speed of abattoirs in Europe and Brazil compared to that in US and Canada may contribute to different levels of SIV contamination*.” How?

Slower line speed of abattoirs in Europe and Brazil may affect to have higher residence time during the collection period and therefore more time for potential contamination of blood with SIV at the slaughter line.

We changed this paragraph in the revised version of the manuscript as following:

*“…It is speculated that slower line speed of abattoirs in Europe and Brazil compared to that in US and Canada resulting in longer time for blood collection that may contribute to increased levels of SIV contamination*.”

Line #338. “…The authors have read the journal's policy and the authors of this manuscript have the following competing interests: EB, CR, and JPolo are employed by APC Europe, S.L.U.” JPujols?

Dr. Joan Pujols is a researcher of IRTA-CReSA and his never has been employed of APC Europe, S.L.U. or APC companies.

Line #584. Table 3. The reviewer #1 is asking why the range of the values of PEDV, PPV and PPRS-US are negative.

We appreciate the comment from reviewer #1. As indicated in the title of the Table 3, these values are expressed in log_10_ and therefore a negative log_10_ values do not mean that the virus particle is negative, only that is less than 1 particle/g of liquid plasma. For example, log_10_ -1.76 TCID_50_/ g liquid plasma in fact, means 0.017 TCID_50_/g liquid plasma

**Reviewer #2:** This is a well-written manuscript that contributes greatly to the literature and warrants publication. However, there is heavy discussion and conclusions that the estimated level of contamination was very low or low, whereas the data suggest otherwise. There was no prior establishment of what constitutes 'high' 'moderate' or 'low' levels of contamination, so these conclusions are entirely that of the authors, which have a conflict of interest in the data interpretation to be low. In reality, up to 100% contamination and relatively low Ct (below that of the established infectious dose) suggest there is risk in this ingredient, but the abstract, discussion, and conclusions try to convince the reader otherwise. It would be more appropriate to recognize there are a high number of positive samples with Ct below the known infectious dose, but that it is likely these are nonviable gene fragments that were inactivated during the manufacturing process.

We appreciated the comment received from the reviewer #2. It is true that the term high, moderate, or low levels of contamination is not defined and is subjective for each expert in the field. However, from the authors, having average values of log_10_ 0.1 for PEDV, 1.56 for PCV-2, -1.4 for PRRSV US strain or 0.17 for PRRSV EU strain are relatively low, especially when compare with values of these viruses as the peak of viremia. Importantly, those values are considered low from the point of view of their potential infectiousness in an animal model. For example, 5.6 × 10^1^ TCID_50_/g (1.75 log_10_ TCID_50_/g) was the minimum PEDV dose in feed to be infective for pigs (Schumacher et al., 2016, Am J Vet Res 77(10):1108-13). For PRRSV USA, a 2-mL inoculum containing 10^1^ fluorescent foci units (equivalent to TCID_50_) of virus per milliliter was found sufficient to achieve infection by either route, but it was the very minimal dose to get it (Yoon et al., 1999 Vet Res 30(6):629-38). Therefore, all values obtained in plasma at slaughter should be considered within the low range of viral loads, most of them below the infectiousness levels as demonstrated when using one of the most susceptible routes of inoculation, the intraperitoneal one (Blázquez et al., 2019 Vet Microbiol 239:108450). Moreover, the likelihood of infection through the oral route is usually much lower compared to other routes such as drinking water or intranasal/oropharyngeal exposure as demonstrated with ASFV for example (Niederwerder et al., 2021 AnimaIs 11, 792). Therefore, all together would point out to low levels of contamination, also assuming this can be considered a subjective appraisal.

In any case, throughout the manuscript we have tried to eliminate this ambiguity of low or high levels of contamination. You can find these changes in the revised version of the manuscript.

Finally, although it is true that some co-authors have conflict of interest, all the data from the study is readily available in tables and supplementary material for the readers and it cannot be underestimated that other co-authors signing this manuscript are worldwide very well-recognized virology researchers in the field. Therefore, the authors consider that there is no bias with the information provided in this manuscript.
