# Supplementary material for: Estimated quantity of swine virus genomes based on quantitative PCR analysis in spray-dried porcine plasma samples collected from multiple manufacturing plants
Source: PLoS One. 2022 May 23;17(5):e0259613. doi: 10.1371/journal.pone.0259613 (PMC9126402; doi:10.1371/journal.pone.0259613)
Supplement: Attachment — Submitted filename: Response to reviewers-2.docx [file pone.0259613.s005.docx]

Additional Editor Comments (if provided):

Thank you for addressing the reviewers comments. I ask that you fix the significant figures and add errors for your stock titers.

For all of the titers given in the methods, the significant figures are too high and errors are needed. Example, it says for PRRS (3268 EU strain) that the final titer was 10^5.48 TCID50/ml and done in triplicate. An error needs to be associated with the final titer and then the significant digits are so that there is only one significant digit in the error. So, if the error is 10^0.5, then the titer is 10^5.5 ± 10^0.5.

The authors appreciated the suggestion from the Academic Editor. We have included in the new version of the manuscript the error for the titers in the inoculum. As example the new reported information for the PRRS EU strain (3268EU) inoculum is as follow: *”… final titer of 10^5.5±0.2^ TCID50/mL.”*

The error should also be fixed in Table 2 and Table 3. The error should have one significant digit and this will tell you how many digits you can use in the number reported. See <https://www.ruf.rice.edu/~bioslabs/tools/data_analysis/errors_sigfigs.html>. Error and significant figures need also be fixed in the supplemental data.

The authors really appreciated the help from the Academic Editor to clarify the request and provide the significant digit for all data included in Table2 and Table 3 and Supplementary Table 1 and Suplementary Table 2. We are deeply in debt for her willing to review all the data in advance to the new submission.

The new revised version of the manuscript includes the correct format for all the data in the mentioned tables.
